# Neurophysiological sex differences in mental rotation in young adults: a narrative review of event-related potentials

**DOI:** 10.3389/fnhum.2026.1777556

**Published:** 2026-09-03

**Authors:** Plamenka Nanova

**Affiliations:** Department of Cognitive Psychophysiology, Institute of Neurobiology, Bulgarian Academy of Sciences (BAS), Sofia, Bulgaria

**Keywords:** ERP, gender differences, healthy adults, mental rotation, sex differences

## Abstract

Sex differences in mental rotation represent one of the most consistently reported behavioural sex differences. Identifying the neurophysiological mechanisms underlying spatial cognition may contribute to a more comprehensive understanding of cognitive processing in males and females. The present review synthesises evidence from event-related potential (ERP) studies and identifies sex-related neurophysiological differences across ERP correlates of mental rotation in healthy young adults. Behavioural evidence was also synthesised to examine its relationship to ERP findings. A structured search of PubMed, Scopus, and Web of Science identified studies published between January 1987 and February 2026. Twelve studies met the inclusion criteria, all involving young adult participants. The evidence suggests that young males may show higher mental rotation performance, accompanied by sex-related differences in ERP amplitude, latency, global field power, and topographic patterns. Early and later ERP differences suggest sex-related differences in neurophysiological processing, although the underlying mechanisms remain uncertain. ERP differences may also occur without behavioural effects, suggesting greater sensitivity of neurophysiological measures. The most consistent finding across studies is that behavioural and neurophysiological sex differences become more pronounced as task difficulty increases, particularly with larger rotation angles and more complex, three-dimensional, less familiar, or less nameable stimuli. In contrast, evidence regarding the effects of response format, stimulus presentation, time constraints, and specific cognitive strategies remains limited or inconsistent. Given the limited number of studies, methodological heterogeneity, and small sample sizes, these findings should be interpreted with caution.

## 1 Introduction

A substantial body of research has identified sex-related differences in adult cognitive function. The “social vs. biological” framework proposes that behaviour is shaped by interactions between biological factors and social factors (Halpern, 2012). However, even when neural differences are observed, it is difficult to determine whether they reflect genetic influences, neuroplastic adaptation to the environment, or both.

Sex-related differences are consistently reported in spatial abilities (Halpern, 2012; Hyde and Linn, 1988; de Frias et al., 2006; Hyde, 2014)–visuospatial (de Frias et al., 2006; Lippa et al., 2010) and auditory spatial (Zündorf et al., 2011), with the most pronounced effects observed in mental rotation (MR), particularly for 3D stimuli (Hyde, 2014; Lippa et al., 2010; Silverman et al., 2007; Voyer, 2011; Levine et al., 2016; Kheloui et al., 2021; Shepard and Metzler, 1971). These differences are evident across cultures (Silverman et al., 2007), with moderate to large effect sizes and persistence across the lifespan (Hyde, 2014).

Evidence suggests that sex differences in mental rotation are partly explained by biological factors, including prenatal and postnatal hormonal influences and genetically determined structural and functional brain differences in regions involved in spatial processing (Levine et al., 2016; Nanova et al., 2024; Silverman and Eals, 1992; Eals and Silverman, 1994; Kimura, 1992).

At the same time, psychosocial factors, including stereotypical expectations and experiences, may also substantially contribute to individual and sex-related differences in mental rotation (Halpern, 2012). Everyday visuospatial activities that often differ between the sexes, such as childhood play activities, navigation, sports, exposure to spatially demanding educational activities, video gaming experience, and spatial training, have all been associated with improved spatial performance (Halpern, 2012; Levine et al., 2016; Feng et al., 2007; Podlogar and Podlesek, 2022). In addition, sex-role stereotypes, parental influences, teacher expectations, educational environment, and broader sociocultural factors may affect performance, confidence, and task approach. Therefore, sex differences in mental rotation are likely to reflect complex interactions between biological predispositions and environmental experience.

Despite robust behavioural evidence for sex differences in mental rotation, the neurophysiological mechanisms underlying these differences remain incompletely understood. Clarifying these mechanisms may improve understanding of sex differences in mental rotation and guide future research toward more fine-grained investigations of neural, genetic, and molecular mechanisms.

Electroencephalography (EEG) is a non-invasive electrophysiological method for recording the brain’s electrical activity through scalp electrodes. EEG-derived approaches can be broadly classified into time-domain, frequency-domain, time-frequency, and source-space analyses. Time-domain approaches include event-related potentials (ERPs), which allow identification of sex-related differences across distinct stages of mental rotation processing. Frequency-domain approaches include qEEG (at rest or during task performance), coherence, and hemispheric asymmetry, providing information about oscillatory activity, functional connectivity, and lateralisation (Hoptman and Davidson, 1998; Rescher and Rappelsberger, 1999; Gill et al., 1998; Di Russo et al., 2002). Time-frequency approaches, such as event-related oscillations, characterize stimulus-related changes in oscillatory activity across time and frequency, whereas source-space approaches such as sLORETA estimate cortical generators (Pascual-Marqui, 2002).

The present review focuses specifically on ERPs because their high temporal resolution makes them particularly suitable for examining stage-specific neurophysiological correlates of mental rotation and provides valuable information about the temporal dynamics of sensory, cognitive, and motor processes (Bradley and Keil, 2012; Luck, 2012; Luck, 2014; Polich, 1998; Regan, 1989).

During mental rotation, ERPs reflect early sensory evaluation (100–300 ms after stimulus onset; occipital, parietal, and frontal areas), evaluation of complex features and strategy selection (300–400 ms; frontal areas), mental rotation itself (400–800 ms; centro-parietal areas), which increases with angular disparity and overlaps with the centro-parietal P300, whose positivity decreases as stimulus orientation deviates more (Wijers et al., 1989), and decision and response preparation stages (from 700 ms to response; centro-parietal and frontal areas) (Desrocher et al., 1995; Griksiene et al., 2019). To the best of our knowledge, no review has specifically synthesised ERP evidence on sex differences in mental rotation in healthy adults.

The aim of the present review was to synthesise ERP evidence and identify consistent sex-related neurophysiological differences across ERP correlates of mental rotation in healthy young adults. Behavioural evidence was also synthesised to examine its relationship to ERP findings. Specifically, the review addressed the following questions: (1) Do early and late ERP components differ between the sexes in amplitude and latency? (2) Are there sex-related differences in ERP scalp topography? (3) Are behavioural sex differences consistently observed during mental rotation? (4) How do task characteristics and stimulus properties influence behavioural and neurophysiological sex differences?

## 2 Methods

A structured literature search was conducted in PubMed, Scopus, and Web of Science to identify studies examining ERPs and sex differences in mental rotation published between January 1987 and February 2026. Searches combined the keywords “ERP,” “mental rotation,” “sex differences,” “gender differences,” and “healthy adults.” Because the terms sex and gender are used inconsistently in the literature, both terms were included to maximize study retrieval. However, the present review focuses primarily on biological differences between males and females and therefore uses the term sex throughout the manuscript.

Inclusion criteria were studies on healthy adult humans, full-text articles in English, and reports of ERP data related to mental rotation and sex differences. Eligible studies were required to compare ERP correlates of mental rotation between biological sexes. Behavioural performance was not an inclusion criterion; however, all 12 included studies reported both ERP and behavioural data. Across the literature, sex was operationalized as a binary between-subject factor (males vs. females). Statistical analyses predominantly relied on mixed-design repeated-measures ANOVA (9 studies), supplemented by specialized techniques such as TANOVA, ANCOVA, TANCOVA, and MANOVA. One study (Wegesin, 1998) additionally assessed sexual orientation using the Sexual Orientation Scale (SOS).

Exclusion criteria included studies on children, adolescents, clinical or animal samples, non-empirical papers, and studies without relevant ERP data. Although the initial aim was to include adults aged 18–65 years, the available literature involved only young adults (18–38 years), therefore, the review focuses on this age group.

The initial search was conducted in PubMed in 2024 using broad and more restrictive keyword combinations. Among the PubMed searches, the broadest keyword combination yielded 827 records. Titles, abstracts, and full texts of potentially eligible studies were screened. Ten studies met the inclusion criteria, including one study identified through PubMed “related articles.”

To update the literature, an additional PubMed search was conducted in 2025. Following a reviewer recommendation, complementary searches were also performed in Web of Science and Scopus in 2026 using the same search strategy. These searches yielded 101 and 11 records, respectively, and identified two additional eligible studies, including one recently published article. In total, 12 studies were included in the review, all reported behavioural performance.

Screening and study selection were performed by a single reviewer. As a narrative review, the aim was to synthesise neurophysiological sex effects and methodological trends rather than conduct a meta-analysis (Baethge et al., 2019). Although narrative reviews are more susceptible to selection bias, the structured multi-database search and complementary screening strategies were applied to improve comprehensiveness and minimise potential selection bias.

## 3 Results

The methodological characteristics and findings of the included studies are summarised in Tables 1 and 2.

**Table 1:** Methodological characteristics.

Study	Number (Number F)	Age (years)	Task (Response choices)	Stimulus presentation	Response limit (s)	RA/AD [°]	Dimensionality	Stimulus type (complexity/nameability/familiarity)
Desrocher et al. (1995)	20 (10F)	18–25	Identical /mirror (2)	Simultaneous	4	RA 0– ±180	2D	Letters (L/H/H)
Desrocher et al. (1995)	20 (10F)	18–25	Identical /mirror (2)	Simultaneous	4.5	RA 0– ±180	2D	Complex figures (M/L/L)
Johnson et al. (2002)	24 (12F)	20–37	Normal/mirror (2)	Single	4	RA 0–300	2D	Letters (L/H/H)
Gootjes et al. (2008)	47 (24F)	19–24	Normal/mirror (2)	Single	3	RA 0–300	2D	Letters (L/H/H)
Beste et al. (2010)	82 (55F)	19–28	Normal/mirror (2)	Single	Unlimited	RA ±30– 150	2D	Letters (L/H/H)
Mikhailova et al. (2012)	19 (10F) 17 (8F)	23–25	Identification (4)	Single	4	±0–90	2D	Drawings (H/H/H)
Pellkofer et al. (2014)	122 (62F)	19–38	Identical /mirror (2)	Simultaneous	Unlimited	AD ±30–150	2D	Polygons (M/L/L)
Wang (2026)	68 (35F)	18–23	Identification (2)	Sequential	12	45–315	2D	Segmented objects (H/H/H)
Wegesin (1998)	40 (20F)	19–31	Identical/mirror (1)	Simultaneous	6	AD 0, 60–180	3D	Blocks (H/L/L)
Yu et al. (2009)	24 (12F)	20–28	Identical/mirror (2)	Simultaneous	4	AD 50, 100	3D	Blocks (H/L/L)
Griksiene et al. (2019)	63 (34F)	20–27	Identical/mirror (2)	Sequential	6.5	AD 25–150	3D	Blocks (H/L/L)
Ruggeri et al. (2020)	55 (26F)	23 (mean)	Identical/mirror	Simultaneous	10	Not specified	3D	Blocks (H/L/L)
Jansen et al. (2020)	58 (27F)	17–27	Same/mirror (2)	Simultaneous	Self-paced	AD 0–180	3D	Cubes (H/L/L)
Jansen et al. (2020)	58 (27F)	17–27	Same/mirror (2)	Simultaneous	Self-paced	AD 0–180	3D	Human figures/postures (M/M/M)

F, females; RA, rotation angle; AD, angular disparity; L, low; M, moderate; H, high; *small sample size.

**Table 2:** Results.

Study	Sex differences in			
	Early ERPs	Rotation ERPs	Topography	Behaviour
Desrocher et al. (1995) ^*^	F: ↑ N4 amp; ↓N4 lat	F: ↑ P3 amp	No	F: more practice to criterion
Johnson et al. (2002) ^*^		M: right parietal pos; F: left/right parietal (hand-dependent)	M: right parietal; F: left/right parietal (hand-dependent)	Mixed acc
Gootjes et al. (2008)	F: ↑ midline amp	M: earlier parietal effect	No	M: ↓ RT (at high angles)
Beste et al. (2010)	No	No		No
Mikhailova et al. (2012) ^*^	M: ↑ parietal P1 amp (angle-related)		M: ↑ parietal	No
Pellkofer et al. (2014)			M: bilateral centro-parietal; F: left centro-parietal	M: ↑ acc; ↓ RT (AD-related)
Wang (2026)	F: ↑ fronto-central amp		F: ↑ fronto-central	M: ↓ RT
Wegesin (1998)		M: ↑ parietal–temporal late slow-wave negativity (trend)		M: ↑ acc
Yu et al. (2009) ^*^	F: ↑ right frontal negativity	No	F: ↑ right frontal	M: ↑ acc (100° AD)
Griksiene et al. (2019)	F: ↑ GFP	M: ↑ left frontal; F: ↑ GFP and parietal positivity	M: left frontal; F: parietal	M: ↑ acc (100° AD); No RT or RT slope differences
Ruggeri et al. (2020)	M: bilateral occipital pos; F: central-frontal neg		M: bilateral occipital; F: central-frontal	M: ↑ acc
Jansen et al. (2020)	No	F: ↑ frontal negativity	F: ↑ frontal	No

^*^Small sample size; F, females; M, males; ↑ – increased; ↓ – decreased; amp, amplitude; lat, latency; GFP, global field power; pos, positivity; neg, negativity; acc, accuracy.

## 4 Discussion

### 4.1 Main results

This review examines the neurophysiological basis of one of the most robust behavioural sex differences - mental rotation. Although psychometric research is extensive, neurophysiological studies remain limited (Griksiene et al., 2019). All included studies involve young adults, and four used small samples, limiting generalisability and requiring cautious interpretation (Section 4.6).

Behavioural sex differences were reported in 9 of 12 studies, typically showing higher accuracy (5 studies) and faster responses (3 studies) in males, particularly in 3D tasks (4 of 5 studies). At the neurophysiological level, 11 of 12 studies reported sex-related differences across early and late ERP components and topographic patterns. Overall, males tended to show higher performance with lower amplitudes or distinct activation patterns, whereas females more often showed higher amplitudes and more extensive activation patterns. Two studies reported neurophysiological differences without behavioural effects (Desrocher et al., 1995; Jansen et al., 2020), suggesting that ERPs may reveal subtle sex-related differences not detected behaviourally.

### 4.2 Early processing ERPs

The reviewed studies suggest that sex differences may emerge during early sensory and perceptual processing, before mental rotation itself (Desrocher et al., 1995; Griksiene et al., 2019; Gootjes et al., 2008; Mikhailova et al., 2012; Wang, 2026; Yu et al., 2009; Ruggeri et al., 2020). Women often showed larger amplitudes of early ERP components and, in some studies, shorter latencies, suggesting sex-related differences in early sensory and perceptual processing (Desrocher et al., 1995; Gootjes et al., 2008; Wang, 2026; Ruggeri et al., 2020). These differences may be related to processing efficiency, neural recruitment, attention allocation, or task engagement. However, the available evidence is insufficient to distinguish among these alternative interpretations.

### 4.3 Rotation related ERPs

During the rotation-related stage, females generally show higher global field power together with larger P3 amplitudes and greater frontal negativity (Desrocher et al., 1995; Griksiene et al., 2019; Ruggeri et al., 2020; Jansen et al., 2020). These findings may reflect differential neural recruitment during mental rotation, greater cognitive effort, compensatory processing, or differences in cognitive strategy. However, the available evidence does not allow these alternative explanations to be distinguished. Increased frontal activity in females (Ruggeri et al., 2020; Jansen et al., 2020) may further suggest greater involvement of processes supporting visuospatial working memory. In contrast, males tend to show reduced parietal amplitudes and lower overall brain activity during rotation, sometimes despite comparable or better behavioural performance. Although this pattern has been interpreted as potentially consistent with the neural efficiency hypothesis (Griksiene et al., 2019; Neubauer and Fink, 2009), ERP amplitude differences alone do not directly establish neural efficiency. In addition, shorter latencies of late ERP components in males may indicate earlier and faster mental rotation processing, potentially contributing to faster behavioural responses (Gootjes et al., 2008). However, limited evidence and methodological heterogeneity preclude firm conclusions regarding the underlying neural mechanisms.

### 4.4 Topographic distribution

Topographic sex-related differences were identified in 8 of 12 studies (Griksiene et al., 2019; Johnson et al., 2002; Mikhailova et al., 2012; Pellkofer et al., 2014; Wang, 2026; Yu et al., 2009; Ruggeri et al., 2020; Jansen et al., 2020). These topographic differences may partly reflect differences in cognitive strategy (Hugdahl et al., 2006). The most commonly proposed distinction is between holistic and analytic processing, with men more likely to rotate stimuli as whole configurations and women relying more on piecemeal processing (Hugdahl et al., 2006; Heil and Jansen-Osmann, 2008; Zawadska, 2022). Other proposed sex differences include visual versus verbal, top-down versus bottom up, and response-selection strategies (Wegesin, 1998; Hugdahl et al., 2006; Heil and Jansen-Osmann, 2008; Ofte and Hugdahl, 2002; Mochizuki et al., 2019; Semrud-Clikeman et al., 2012; Butler et al., 2006; Zawadska, 2022; Hirnstein et al., 2009; Glück and Fabrizii, 2010; Brandner and Devaud, 2013). These strategies may differentially engage the hemispheres (Pletzer et al., 2014) and place different demands on working memory (Griksiene et al., 2019; Zawadska, 2022). However, evidence for sex-related cognitive strategies remains inconsistent because most studies did not assess strategy use directly; consequently, ERP findings provide only indirect evidence. Griksiene et al. (2019) reported no sex differences in reaction time slopes despite clear neurophysiological differences, whereas Wang (2026) observed ERP differences under task conditions designed to minimise strategy differences. Therefore, strategy-based interpretations remain speculative and should be considered alongside alternative explanations, including stimulus familiarity, working memory demands, cognitive resources, prior spatial experience, and differences in neural recruitment during task performance.

Parietal lateralisation patterns suggest greater right-hemisphere involvement in males and more left-lateralised processing in females (Johnson et al., 2002; Pellkofer et al., 2014). Anterior-posterior differences include greater frontal engagement in females and stronger parietal-occipital activation in males (Mikhailova et al., 2012; Pellkofer et al., 2014; Wang, 2026; Yu et al., 2009; Ruggeri et al., 2020; Jansen et al., 2020). These patterns may reflect differences in neural recruitment, cognitive effort, or visual cortical structure (Butler et al., 2006; Amunts et al., 2007), but remain tentative (Ramos-Loyo et al., 2022).

Although cognitive strategy has received considerable attention, biological factors may also contribute to topographic sex differences. Both organizational effects of prenatal and pubertal hormonal influences and activational effects of circulating sex hormones in adulthood have been proposed to influence visuospatial performance and its neural correlates. Developmental EEG studies suggest that sex-related neural differences become more apparent after puberty, consistent with a contribution of pubertal hormonal maturation (Roberts and Bell, 2000; Roberts and Bell, 2003). In adults, fluctuations in estradiol and progesterone across the menstrual cycle have been associated with changes in mental rotation performance (Hausmann et al., 2000) and electrophysiological responses (O’Reilly et al., 2004), whereas testosterone has been positively associated with spatial performance (Hausmann et al., 2000). Consistent with these findings, Griksiene et al. (2019) reported that higher estradiol levels tended to be associated with lower accuracy and higher progesterone levels with longer response times, whereas no associations with testosterone were observed. However, evidence directly linking hormonal status to ERP correlates of mental rotation remains limited.

### 4.5 Task and stimulus characteristics

Neurophysiological sex-related differences were observed across early and late ERP components and topographic patterns (11/12 studies), but varied depending on task demands and stimulus characteristics.

#### 4.5.1 Task design

Task design variables, including task type, stimulus presentation, response format, and time limits, influence mental rotation demands. Simpler normal/mirror judgments, mainly used in letter rotation paradigms, rely more on internal representations and generally reveal fewer sex differences, reported in about half or fewer studies. In contrast, identical/mirror and same/mirror judgments require direct stimulus comparison, imposing greater visuospatial transformation and working memory demands, and more consistently reveal behavioural and neurophysiological sex differences across most studies.

Stimulus presentation further modulates processing. Simultaneous presentation allows direct comparison while reducing working memory load, emphasizing visuospatial transformation. By contrast, sequential presentation requires maintaining the first stimulus in working memory, increasing cognitive load and executive demands. Although based on limited evidence (Griksiene et al., 2019), comparison with studies using simultaneous presentation (Wegesin, 1998; Yu et al., 2009; Ruggeri et al., 2020; Jansen et al., 2020) suggests that sequential 3D block rotation tasks may evoke more pronounced left frontal activity in males, possibly reflecting working memory involvement. Tasks involving internal representations, such as single-letter paradigms, may additionally engage long-term memory. However, no clear conclusions can be drawn for letter-based tasks. Response format varied little, because most studies used two-choice button responses, preventing meaningful analysis of sex effects.

Time limits may influence behavioural and neurophysiological responses. Under greater time pressure, performance likely relies more on rapid, automatic visuospatial transformations, whereas longer or self-paced conditions may allow engagement of controlled, strategy-based processing. For example, under a short 3-s response window, males showed faster responses at larger angles and earlier rotation-related activity (Gootjes et al., 2008). However, given the limited number of studies and small sample sizes, no meaningful conclusions can be drawn regarding time constraints.

#### 4.5.2 Rotation demands

Rotation angle and angular disparity are major determinants of task difficulty in mental rotation. Increasing angular disparity is consistently associated with longer reaction times, lower accuracy, prolonged ERP latencies, greater amplitudes, and increased parietal activity, reflecting higher cognitive demands. Behavioural sex differences become more evident at larger rotation angles, with males generally showing higher accuracy and occasionally faster response times (Griksiene et al., 2019; Johnson et al., 2002; Gootjes et al., 2008; Pellkofer et al., 2014; Yu et al., 2009).

Mental rotation tasks employ both 2D and 3D stimuli, which differ in cognitive demands. Two-dimensional stimuli, particularly letters, are generally less complex, allowing faster processing and fewer behavioural sex differences (4/7) (Johnson et al., 2002; Gootjes et al., 2008; Pellkofer et al., 2014; Wang, 2026). Nevertheless, ERP differences are still observed in several 2D studies (5/7) (Desrocher et al., 1995; Johnson et al., 2002; Gootjes et al., 2008; Mikhailova et al., 2012; Wang, 2026), including larger amplitudes and shorter latencies during early processing, greater amplitudes in females during rotation, and earlier rotation-related activity in males. Topographic differences are also reported (Johnson et al., 2002; Mikhailova et al., 2012; Pellkofer et al., 2014; Wang, 2026), although evidence for consistent strategy differences remains limited. In contrast, three-dimensional stimuli (e.g., block figures) require depth processing and impose greater visuospatial and working memory demands. Accordingly, 3D tasks more consistently reveal behavioural (4/5) (Griksiene et al., 2019; Wegesin, 1998; Yu et al., 2009; Ruggeri et al., 2020) and neurophysiological sex differences, including more pronounced amplitudes, higher global field power, and more consistent topographic variation (4/5 studies).

#### 4.5.3 Stimulus characteristics

Stimulus complexity, familiarity, and nameability further modulate mental rotation performance. Simple, familiar stimuli, particularly letters, are a relative exception to commonly reported sex-related effects. Their familiarity and potentially more holistic processing may reduce task difficulty and partly explain reduced or absent sex differences. Overall, males and females often show comparable performance in letter rotation, although females may require more practice to reach high accuracy, whereas faster male responses typically emerge at larger rotation angles (Desrocher et al., 1995; Johnson et al., 2002; Gootjes et al., 2008; Beste et al., 2010). Neurophysiological differences may be present, particularly during early processing and rotation, but topographic differences are less consistently observed (Desrocher et al., 1995; Johnson et al., 2002; Gootjes et al., 2008; Beste et al., 2010).

In contrast, more complex, less familiar, and less nameable stimuli (e.g., polygons and 3D block figures) more consistently reveal behavioural and neurophysiological sex differences. These tasks are associated with higher male accuracy (up to 4/5 studies), larger amplitudes and global field power in females, and more pronounced topographic differences (4/5 studies). Such patterns are often interpreted as strategy-related, although differential neural engagement cannot be excluded.

Overall, the most consistent finding is that sex-related behavioural and neurophysiological differences become more pronounced as task difficulty increases, particularly with larger rotation angles, higher stimulus complexity, and 3D, less familiar, or less nameable stimuli. In contrast, the effects of response format, stimulus presentation, time constraints, and specific cognitive strategies remain limited or inconsistent.

### 4.6 Methodological issues

A limitation is that 4 of 12 studies included small samples (8–12 participants per group) (Desrocher et al., 1995; Johnson et al., 2002; Mikhailova et al., 2012; Yu et al., 2009), reducing statistical power, reliability, and the robustness of the findings (Button et al., 2013). Low statistical power increases the risk of missing true effects and producing unstable or non-replicable results, whereas larger samples (>20 participants per group) provide more reliable and generalizable estimates (Cohen, 1992). Together with substantial methodological heterogeneity, small sample sizes reduce comparability and require cautious interpretation of the findings. In addition, screening and study selection were performed by a single reviewer, which may have increased the risk of selection bias. Future research should consider potential confounding factors. These include task-related characteristics such as stimulus features, complexity, rotation angle, instructions, presentation mode, time constraints, and statistical power (Jansen et al., 2020; Ramos-Loyo et al., 2022). Individual and neurobiological factors, including hormonal influences, age, handedness, personality, cognitive strategies, and stimulus salience, may contribute to performance variability. Prior spatial training and experience acquired through education (e.g., geometry or engineering) or activities such as video gaming, sports, driving, map use, and cognitive training may also influence performance. An additional limitation is that all included studies involved young adults, restricting the generalizability of the findings. Therefore, the conclusions may not extend to older adults or adolescents, in whom aging-related, developmental, or educational factors may influence mental rotation performance and neurophysiological correlates.

A further limitation is the predominant focus on time-locked ERP measures, which provides limited information about ongoing frequency-specific and connectivity-related brain dynamics. Future studies integrating ERP with complementary EEG approaches, including qEEG, ERO analyses, functional connectivity, and source-space methods, may provide a more comprehensive understanding of sex differences in mental rotation.

## 5 Conclusion

The available evidence suggests that young males may show higher mental rotation performance than females, accompanied by sex-related differences in ERP measures, including amplitude, latency, global field power, and topographic patterns. Early ERP differences may reflect sex-related differences in early sensory and perceptual processing, potentially related to processing efficiency, neural recruitment, attention allocation, or task engagement. Differences in later ERP components may reflect differential neural recruitment, cognitive effort, compensatory processing, or differences in cognitive strategies. However, the available evidence is insufficient to distinguish among these alternative interpretations. ERP differences may also occur without behavioural effects, suggesting greater sensitivity of neurophysiological measures. The most consistent finding across studies is that behavioural and neurophysiological sex differences become more pronounced as task difficulty increases, particularly with larger rotation angles and more complex, three-dimensional, less familiar, or less nameable stimuli. In contrast, evidence regarding the effects of response format, stimulus presentation, time constraints, and specific cognitive strategies remains limited or inconsistent. Given the limited number of studies, methodological heterogeneity, and small sample sizes, these findings should be interpreted with caution. Future research should employ larger samples, standardized mental rotation paradigms, longitudinal designs, and more diverse populations across the lifespan and cultures to improve comparability across studies and clarify developmental changes in sex differences and the underlying neurophysiological mechanisms. Studies investigating interactions between biological (e.g., hormonal) and experiential (e.g., spatial training, education, lifestyle) factors may provide a more comprehensive understanding of these sex differences.
